# Distal coronary sinus–left atrial fistulous communication uncovered during atrial fibrillation ablation

**DOI:** 10.1093/ehjcr/ytag030

**Published:** 2026-01-24

**Authors:** Yoshihiro Sobue, Tomoyuki Ohno, Eiichi Watanabe, Hideo Izawa

**Affiliations:** Department of Cardiology, Fujita Health University Bantane Hospital, 3-6-10 Otobashi, Nakagawa-ku, Nagoya, Aichi 454-8509, Japan; Department of Radiation, Fujita Health University Bantane Hospital, 3-6-10 Otobashi, Nakagawa-ku, Nagoya, Aichi 454-8509, Japan; Department of Cardiology, Fujita Health University Bantane Hospital, 3-6-10 Otobashi, Nakagawa-ku, Nagoya, Aichi 454-8509, Japan; Department of Cardiology, Fujita Health University School of Medicine, 1-98 Dengakugakubo, Kutsukake-cho, Toyoake, Aichi 470-1192, Japan

**Keywords:** Coronary sinus anomaly, Unroofed coronary sinus, Partial anomalous pulmonary venous connection, Left atrial appendage, Atrial fibrillation, Case report

## Abstract

**Background:**

Congenital anomalies of the coronary sinus (CS) are rare and often overlooked on standard imaging. Among these, the unroofed coronary sinus (URCS) is the most recognized, typically involving the proximal CS and frequently associated with a persistent left superior vena cava. In contrast, fistulous communications between the distal CS and the left atrium (LA) are exceptionally uncommon. Their recognition is important during atrial fibrillation (AF) ablation, where unanticipated channels may complicate catheter manipulation and lesion delivery.

**Case summary:**

A 76-year-old woman with recurrent paroxysmal AF underwent catheter ablation. A lumen-equipped multipolar catheter was positioned in the CS, and transseptal access to the LA was achieved. During electroanatomical mapping with a high-density catheter, entanglement with the CS catheter suggested an anomalous connection. Contrast injection through the CS catheter opacified the left atrial appendage (LAA), and selective angiography revealed simultaneous filling of the LAA and the left inferior pulmonary vein (LIPV), confirming a fistulous tract between the distal CS, LAA, and LIPV. Pulmonary vein isolation was completed without complication. Retrospective review of pre-procedural computed tomography demonstrated a connecting vein from the distal CS to the LA at the LAA–LIPV ridge, corroborating the procedural findings.

**Discussion:**

This case highlights a rare distal CS–LA fistulous communication involving the LAA and LIPV. It broadens the anatomical spectrum between URCS and partial anomalous pulmonary venous connection. Recognition of these anomalies is critical for procedural safety during AF ablation, underscoring the value of multimodality imaging and intra-procedural venography.

Learning points

- Distal CS–LA communications should be considered when unexpected catheter entrapment or atypical LA opacification occurs during AF ablation.
- Targeted coronary sinus venography helps distinguish distal CS–LA fistulas from URCS or vein of Marshall variants.

## Introduction

The coronary sinus (CS) originates embryologically from the left horn of the sinus venosus.^1^ In normal anatomy, the CS courses along the posterior atrioventricular groove and drains into the right atrium through a discrete ostium located between the inferior vena cava and the tricuspid annulus.

Congenital anomalies of the CS are uncommon, with an estimated prevalence of approximately 0.1%–1.0% in the general population.^1^ Reported variants include persistent left superior vena cava (PLSVC), unroofed coronary sinus (URCS), coronary sinus ostial atresia, and CS diverticulum. Unroofed coronary sinus represents the rarest form of atrial septal defect, characterized by partial or complete absence of the CS wall, usually involving its proximal segment and frequently associated with PLSVC.^2–4^ Although URCS represents the most common congenital anomaly associated with communication between the proximal CS and the left atrium (LA), fistulous connections arising from the distal CS to the LA remain little known and poorly characterized. Herein, we describe a rare and anatomically distinctive case of a distal CS fistulous connection with both the left atrial appendage (LAA) and the left inferior pulmonary vein (LIPV), identified incidentally during catheter ablation for atrial fibrillation (AF).

## Summary figure

**Figure:**
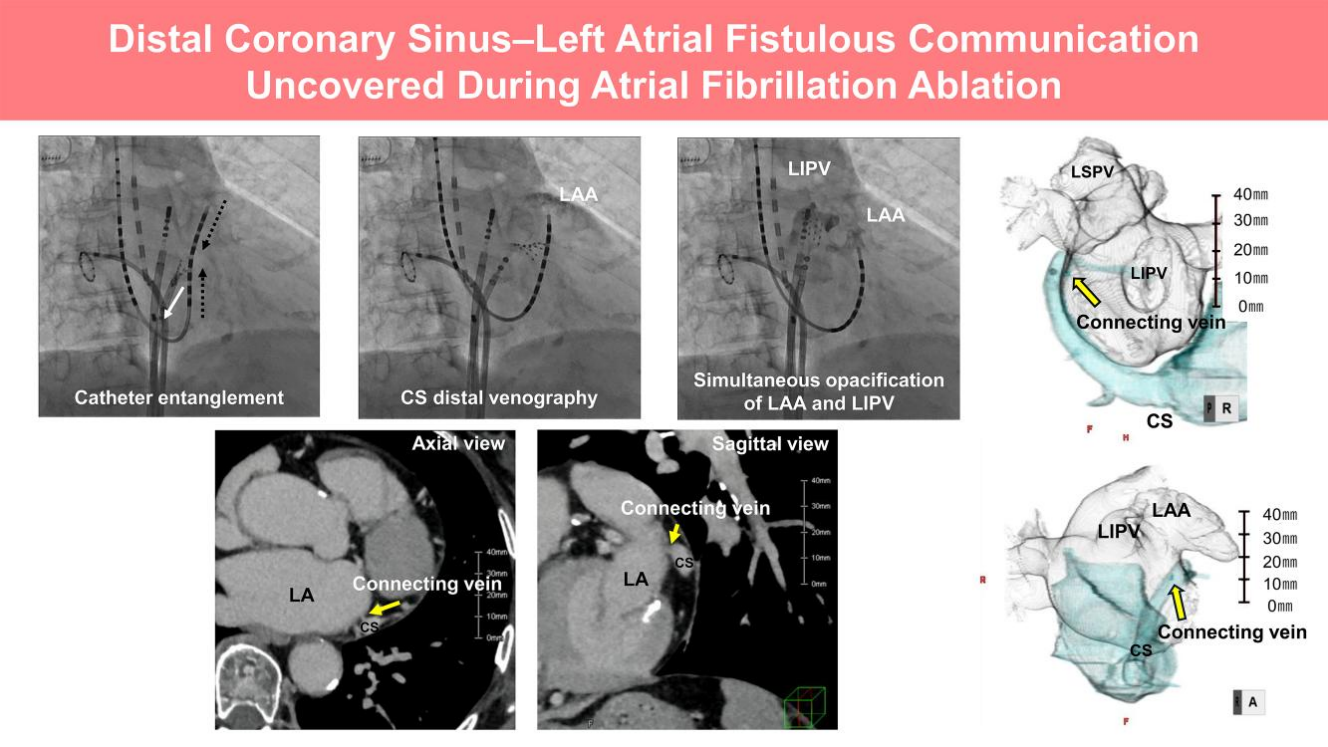
Distal CS–left atrial fistulous communication identified during atrial fibrillation ablation. Coronary sinus distal venography showed opacification of the LAA with simultaneous LIPV filling, and CT (axial and sagittal views) confirmed the connecting vein.

## Case presentation

A 76-year-old woman with recurrent, symptomatic paroxysmal atrial fibrillation presented with palpitations. Transthoracic echocardiography showed a preserved left ventricular ejection fraction (62%) and a mildly enlarged LA (diameter 37 mm; volume index 44.8 mL/m²). She was admitted for catheter ablation. After venous access was obtained, a lumen-equipped multipolar catheter (BeeAT, Japan Lifeline, Tokyo, Japan) was introduced into the CS for reference and pacing. Subsequently, a transseptal puncture was performed under fluoroscopic and electroanatomical guidance. Thereafter, an anatomical and activation map of the LA was systematically acquired using a high-density multipolar mapping catheter (Advisor™ HD Grid, Abbott, Abbott Park, IL, USA).

**Figure 1:**
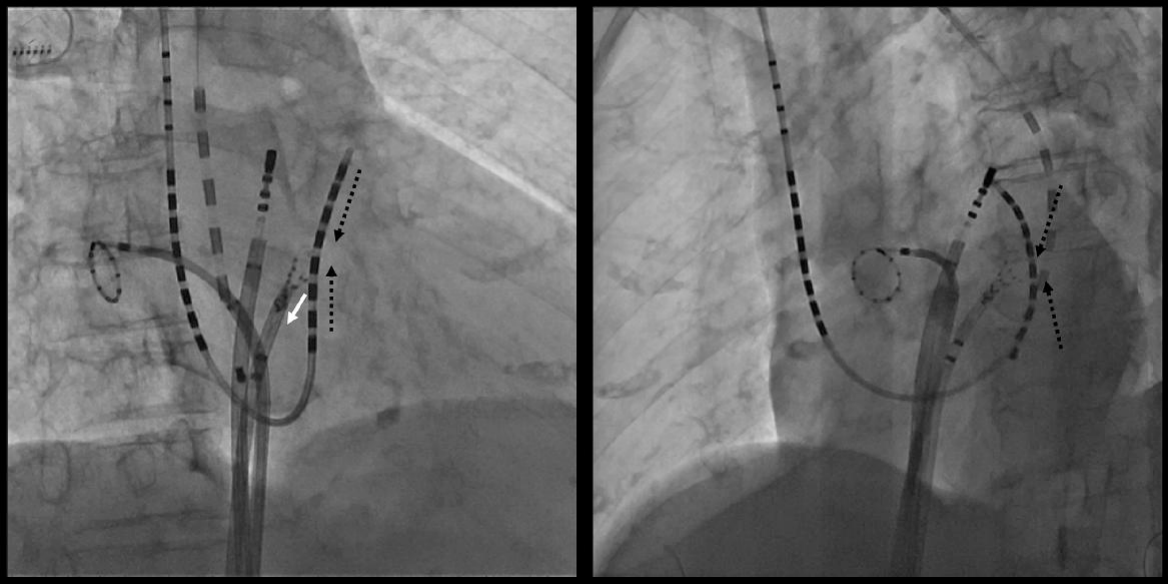
Mechanical entanglement of an HD Grid catheter in the left atrium with a lumen-equipped BeeAT catheter in the distal coronary sinus during mapping. The white arrow indicates the direction of traction applied by the HD Grid catheter, while the black dotted line denotes the resulting force vector pulling the coronary sinus catheter electrodes.

**Figure 2:**
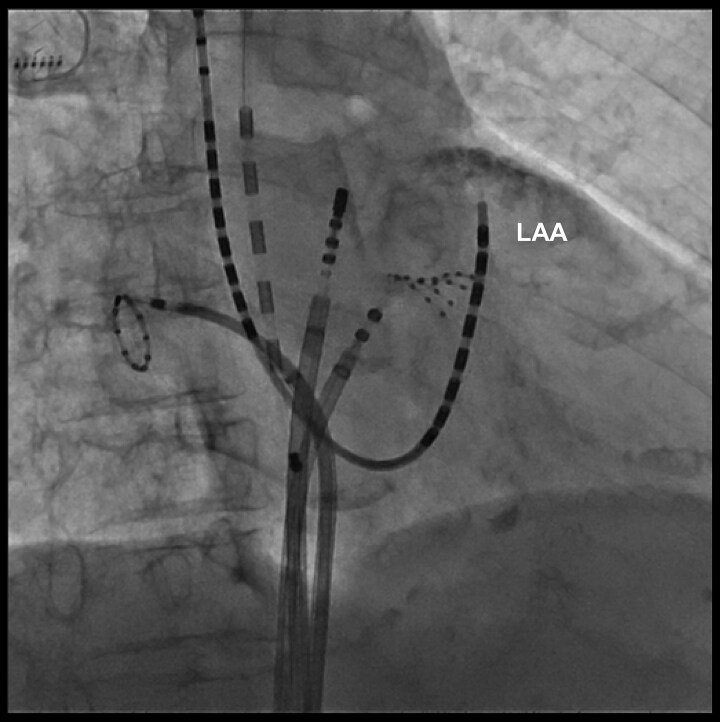
Contrast injection through the distal coronary sinus lumen opacifying the left atrial appendage, indicating a fistulous tract.

**Figure 3:**
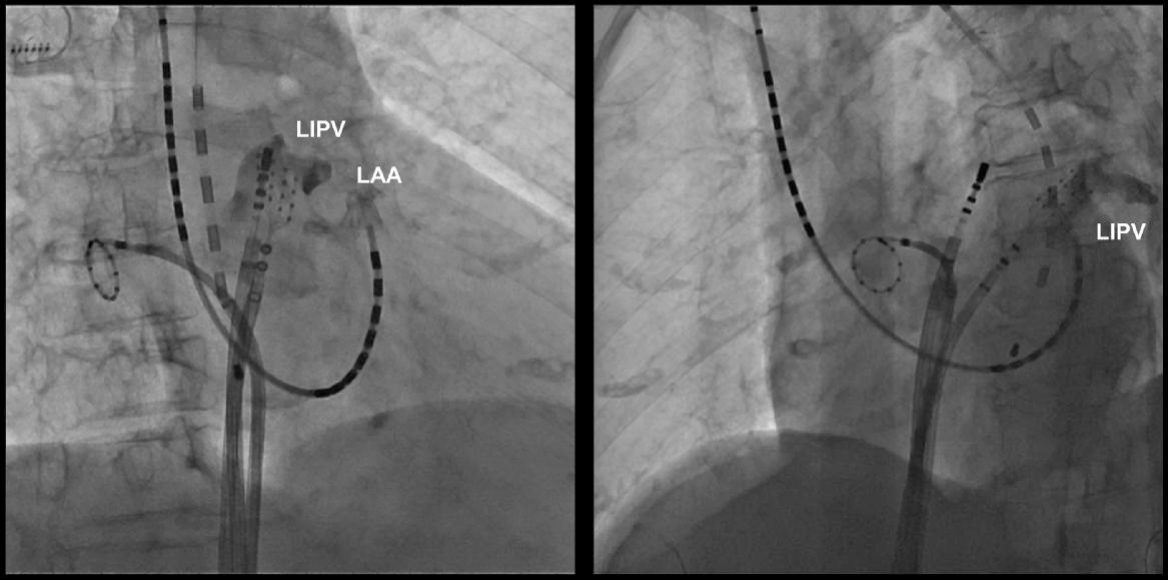
Selective coronary sinus angiography demonstrating simultaneous opacification of the left atrial appendage and the left inferior pulmonary vein.

During the mapping procedure, mechanical entanglement occurred between the HD Grid catheter in the LA and the BeeAT catheter positioned in the distal CS (*Figure 1*; Supplementary material online, *VideoS1*). This raised suspicion of an anomalous communication between the CS and the LA. To confirm this suspicion, contrast medium was injected through the lumen of the BeeAT catheter, opacifying the LAA and demonstrating a fistulous tract (*Figure 2*;Supplementary material online, *Video S2*). For precise delineation of the fistulous site, the CS catheter was repositioned more proximally and selective contrast injection was repeated. This demonstrated simultaneous opacification of the LAA and the LIPV (*Figure 3*;Supplementary material online, *Video S3*), thereby establishing the presence of a fistulous communication between the distal CS, the LAA, and the LIPV.

Electrical isolation of all four pulmonary veins was successfully achieved, and the procedure was completed without complications. Isoproterenol infusion and programmed pacing from both the distal and proximal CS were performed to assess arrhythmogenicity; however, AF was not inducible. Therefore, no ablation was applied to the connecting vein or the CS. At 8-month follow-up, the patient remained in sinus rhythm without AF recurrence. Oral anticoagulation was continued based on a CHA₂DS₂-VASc score of 2. Because the distal CS–LA communication was small, haemodynamically insignificant, and non-arrhythmogenic, no intervention was performed and conservative follow-up was chosen.

**Figure 4:**
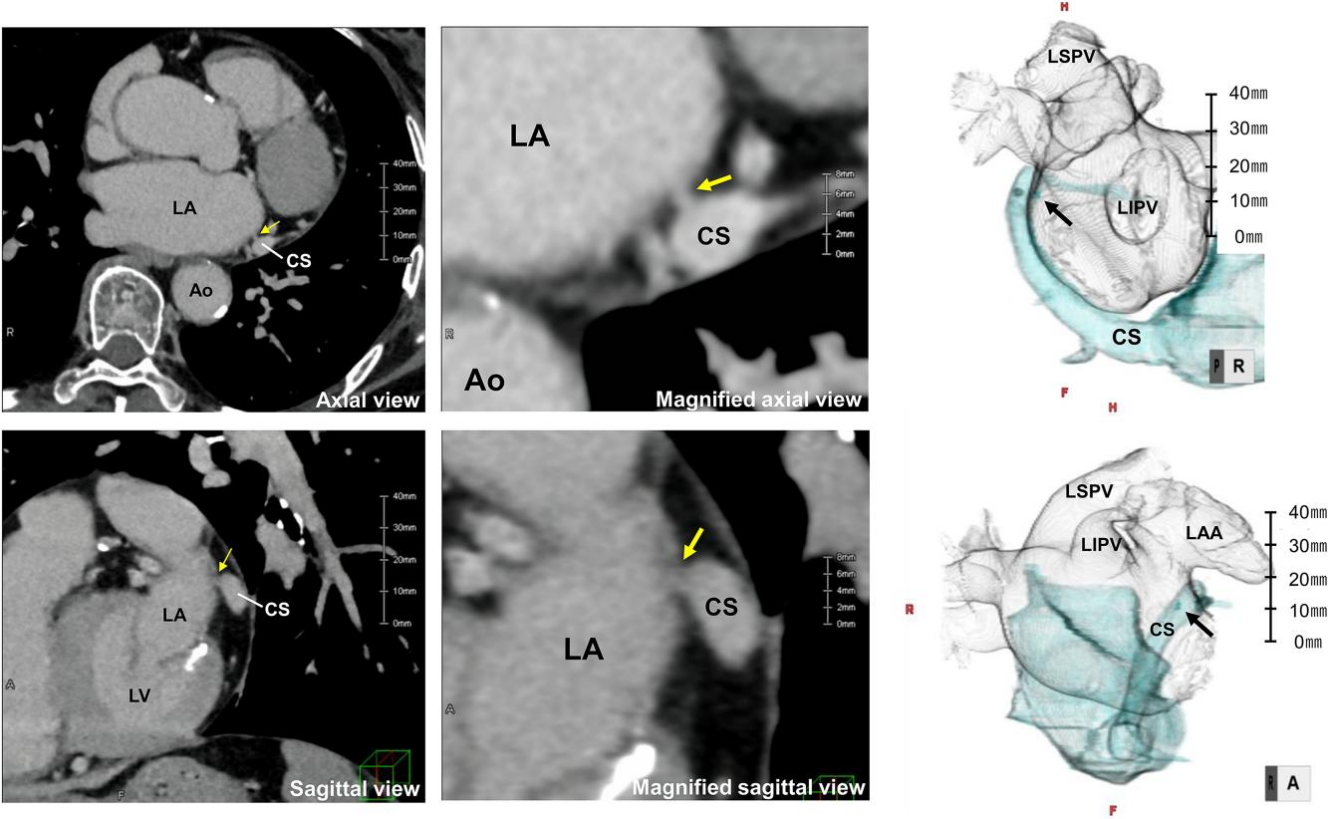
Computed tomography images including axial, magnified axial, sagittal, and magnified sagittal views, as well as a 3D reconstruction, demonstrating a connecting vein arising from the distal coronary sinus and entering the left atrium at the left atrial appendage–left inferior pulmonary vein ridge. Yellow arrows indicate the connecting vein on CT images, and the black arrow highlights the same structure on the 3D reconstruction. Scale bars are shown for reference. LA, left atrium; LAA, left atrial appendage; LIPV, left inferior pulmonary vein; LSPV, left superior pulmonary vein; CS, coronary sinus; Ao, aorta; LV, left ventricle.

Cardiac CT was performed using a 320-detector row scanner (Aquilion ONE, Canon Medical Systems, Japan) with a slice thickness of 0.5 mm during the contrast-enhanced left atrial phase and the delayed phase (90 s after contrast injection). The CT images shown correspond to the delayed phase, which best delineated the fistulous communication. Retrospective review of the pre-procedural contrast-enhanced computed tomography images, which had initially been interpreted as normal, revealed subtle contrast continuity between the CS at the level of the mitral annulus (approximately 1 o’clock in the left anterior oblique projection) and the adjacent left atrial wall, corroborating the intra-procedural diagnosis (*Figure 4*).

## Discussion

We reported a rare distal CS–LA communication uncovered during AF ablation, with the fistulous tract coursing between the LAA and the LIPV. As previous case reports of CS–LA fistulas have predominantly described defects arising from the proximal or mid CS,^5^ communications originating from the distal CS to the LA remain little known and poorly characterized.^6^ From an embryological perspective, these anomalies may be caused by either a distal variant within the spectrum of URCS or, alternatively, a form of partial anomalous pulmonary venous connection draining into the CS.^7^ Recognition of these variants is clinically important, not only for accurate anatomical classification but also because fistulous channels may alter local conduction properties, serve as potential triggers for atrial fibrillation, and complicate catheter manipulation.

Unroofed coronary sinus is a rare congenital anomaly defined by partial or complete absence of the partition between the CS and LA. Based on the extent of unroofing and the presence or absence of a PLSVC, four morphological types with subtypes ‘a’ (with PLSVC) and ‘b’ (without PLSVC) are recognized (*Table 1*).^3^ In the present case, we identified a distal CS–LA communication located at the LAA/LIPV ridge in the absence of an atrial septal defect or a PLSVC. Morphologically, this anomaly corresponds to type IVb in the contemporary URCS classification, defined as an anomalous conduit between the CS and the LA without PLSVC. Although proximal CS–LA communications have been reported,^5,8^ a distal fistulous tract has not previously been demonstrated using both high-resolution 3D mapping and contrast venography. This case therefore represents the first documented distal type IVb URCS, with a connection from the distal CS to the LAA–LIPV ridge.

**Table 1:** Classification of URCS by defect site and association with PLSVC

Type	Complete or partial defect	Defect site of CS	With PLSVC	Without PLSVC
I	Complete	–	Ia	Ⅰb
Ⅱ	Partial	Proximal	Ⅱa	Ⅱb
Ⅲ	Partial	Distal	Ⅲa	Ⅲb
Ⅳ	Partial	Connecting vein	Ⅳa	Ⅳb

CS, coronary sinus, PLSVC, persistent left superior vena cava.

The connecting vein arose from the distal CS and entered the LA at the LAA–LIPV ridge. Although the vein of Marshall typically drains into the proximal to mid CS,^9,10^ the course of this channel overlapped its expected trajectory, suggesting an embryologically related but distinct vestigial variant with a persistent atrial opening. This region can be arrhythmogenic, but no spontaneous or inducible triggers were observed.

Small or incidentally detected URCS variants are generally managed conservatively, with intervention reserved for cases involving significant shunting or associated congenital anomalies. In this patient, the absence of haemodynamic impact or arrhythmogenic substrate justified conservative management with continued anticoagulation based on the CHA₂DS₂-VASc score. Their identification carries important procedural implications, underscoring the need for meticulous imaging and intra-procedural assessment to ensure safe and effective ablation strategies.

## Conclusion

Distal CS–LA fistulous connections represent an exceedingly rare venous anomaly that may reflect either a distal variant of URCS or an embryologic remnant of the VOM. Recognition of these variants is of clinical importance, as their presence has direct implications for safe catheter manipulation and procedural planning and therapeutic strategies in left atrial interventions.

## Lead author biography


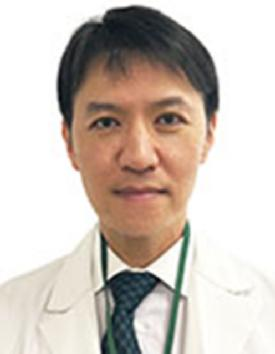


Dr Yoshihiro Sobue is a cardiologist and electrophysiologist at Fujita Health University Bantane Hospital, Nagoya, Japan. He received his MD and PhD from Fujita Health University and has specialized training in cardiac electrophysiology and catheter ablation. His clinical and research interests focus on atrial fibrillation, ventricular arrhythmias, heart failure, and advanced mapping/imaging technologies. He has published widely in peer-reviewed journals and actively contributes as a reviewer and editorial board member for international cardiovascular journals.

## Supplementary Material

ytag030_Supplementary_Data

## Data Availability

All data underlying this article are included within the article and its online Supplementary material.

## Supplementary material

**Consent:** Written informed consent for publication was obtained from the patient in line with the COPE guidance.
